# Functional Inhibition of Natural Killer Cells in a BALB/c Mouse Model of Liver Fibrosis Induced by *Schistosoma japonicum* Infection

**DOI:** 10.3389/fcimb.2020.598987

**Published:** 2020-11-19

**Authors:** Yuan Hu, Xiaoling Wang, Yuhuan Wei, Hua Liu, Jing Zhang, Yujuan Shen, Jianping Cao

**Affiliations:** Key Laboratory of Parasite and Vector Biology, National Institute of Parasitic Diseases, Chinese Center for Disease Control and Prevention, Chinese Center for Tropical Diseases Research, WHO Collaborating Center for Tropical Diseases, Shanghai, China

**Keywords:** natural killer cells, *Schistosoma japonicum*, liver fibrosis, activated receptor, inhibitory receptor

## Abstract

**Background and Aims:**

Schistosomiasis japonica is a widespread human zoonotic disease, and in China, there are many patients with schistosomiasis suffering from liver fibrosis. Many studies have shown that natural killer (NK) cells could reduce the progression of hepatic fibrosis by directly killing hepatic stellate cells (HSCs). However, NK cells could not inhibit the progress of liver fibrosis induced by *Schistosoma japonicum* infection. We aimed to investigate the function of NK cells in schistosomiasis.

**Methods:**

BALB/c mice were infected with S. japonicum cercariae. The receptors and their proportions expressed on NK cells in the liver and spleen from infected mice were detected using flow cytometry. Levels of IFN-γ, perforin, and granzyme of NK cells, and collagen I, III, and α-SMA of hepatic tissue, were detected using quantitative real-time PCR. Changes in cytokine levels in sera were detected using a cytometric bead array. Liver fibrosis was evaluated using hematoxylin and eosin and Masson staining. NK function in the schistosomiasis model was analyzed.

**Results:**

From 2 to 4 weeks post-infection, NK cells were activated, with significantly increased levels of effector molecules (IFN-γ, perforin, and granzyme) that peaked at 4 weeks after infection. The proportion of NK cells increased in the liver and spleen from 6 to 10 weeks post-infection. However, the function of NK cells was inhibited from 6 to 10 weeks post-infection with significantly decreased levels of activated receptors (AR), inhibitory receptors (IR), and effector molecules. The levels of IFN-γ, IL-12, and IL-6 in mouse serum peaked at 6 weeks post-infection, and IL-10 and IL-21 levels peaked at 8 weeks post-infection. Hepatic fibrosis markers increased significantly at 6 weeks after infection.

**Conclusion:**

Our study suggested that NK cells were activated from 2 to 4 weeks post-infection and participated in inflammation in the mouse model. After the S. japonicum laid their eggs, NK cells became inhibited, with decreased levels of both activating and inhibitory NK cell receptors, as well as cytotoxic molecules. In addition, liver fibrosis formed. In mice infected with S. japonicum, the process of liver fibrosis might be alleviated by removing the functional inhibition of NK cells.

## Introduction

Schistosomiasis is an important zoonotic disease. In China, more than 450 counties were endemic for schistosomiasis, which threatened nearly 100 million people (Colley et al., 2014). In recent years, schistosomiasis has been effectively controlled in China. However, there are many patients with schistosomiasis with liver fibrosis, which has reduced the labor force and caused a severe economic burden in China. After infection with *Schistosoma japonicum*, a large number of eggs that release soluble egg antigen (SEA) are deposited in the host liver, which induces cytokines [interleukin (IL)-13, IL-4, and transforming growth factor-beta (TGF-β)] production, activates the signal transducer and activator of transcription 6 (STAT6) signaling pathway, and induces polarization of the Th2 immune response (Neill et al., 2010; Du et al., 2016). These changes promote the activation of hepatic stellate cells (HSCs), which produce large collagen deposits, leading to the formation of egg granulomas and liver fibrosis.

Activation of HSCs plays a central role in the formation of hepatic fibrosis. Quiescent HSCs are activated and then proliferate and differentiate into myofibroblasts, which secrete many collagen fibers. When the rate of collagen synthesis exceeds that of degradation, liver fibrosis occurs (Tsuchida and Friedman, 2017). Natural killer (NK) cells are a type of innate immune cell, and numerous studies have shown that NK cells have anti-fibrotic effects (Yi et al., 2014; Fernández-Álvarez et al., 2015; Fan et al., 2020). NK cells may directly kill the activated HSCs by ligand-receptor binding and degranulation, or by secreting IFN-γ (Gao et al., 2009; Peng and Tian, 2018).

Some studies have shown that NK cells also have an anti-fibrotic effect in schistosomiasis models. NK cells from the livers of mice infected with *S. japonicum* killed HSC-derived myofibroblasts, which negatively regulated liver fibrosis (Hou et al., 2012). The percentage of NK cells increased significantly from the 5th to the 7th week after infection with *S. japonicum*, and in infected mice, NK cells were activated, accompanied by increased surface CD69 expression (Luo et al., 2014). Another study showed that the population of NK cells increased from the 5th to the 6th week post-infection, and a higher percentage of IFN-γ+ NK cells, and a lower percentage of IL-5+ NK cells, were observed in the lungs of infected mice (Cha et al., 2017). However, the specific function of NK cells in the schistosomiasis model remains unclear. The immune responses in mice were significantly different before and after spawning of *S. japonicum* (Wen et al., 2011). Does the role of NK cells change before and after *Schistosoma* spawning?

In the present study, an *S. japonicum* infection BALB/c mouse model was used to investigate the dynamic changes of NK cells proportions, the expression receptor, and effector molecules expression in the livers and spleens from the 2nd to the 10th week post-infection with *S. japonicum*. The characteristics of NK cells in the schistosomiasis mouse model were evaluated.

## Materials and Methods

### Ethics Statement

All experiments involving BALB/c mice were performed according to the recommendations of the Laboratory of Animal Welfare and Ethics Committee (LAWEC) of China. The LAWEC Committee of the National Institute of Parasitic Diseases, Chinese Centre for Disease Control and Prevention approved the protocol (approval ID: IPD 2009–4).

### Mice, Parasites, and Infection

Female BALB/c mice of specific pathogen-free (SPF) grade, 6 weeks old and weighing 20 ± 2 g, were purchased from the Shanghai Slack Laboratory Animal Co., Ltd (Shanghai, China) and raised in an SPF grade animal room in the Institute of Parasitic Disease Prevention and Control, China Center for Disease Control and Prevention. The snail room of our institute provided *S. japonicum* cercariae (mainland China strain). The snails, *Oncomelania hupensis*, came from Jiangxi Province. Forty BALB/c mice were randomly divided into two groups: infected and uninfected. Each mouse in the infected group was infected with 20 ± 2 cercariae *via* their abdominal skin. The experiments were repeated three times, and the number of mice was not less than four per time point for each repetition.

### Isolation of Lymphocytes

BALB/c mice were sacrificed *via* neck dislocation after anesthesia. Liver perfusion in the mice was performed using 1 × Dulbecco’s phosphate-buffered saline (DPBS) (without Ca^2+^ and Mg^2+^) *via* the portal vein. Harvested livers were washed with PBS. The tissue was quickly minced with scissors and placed in 50-ml conical tubes. Tissues were dissociated into single cell suspensions using an Ultra Turrax^®^ Tube Disperser (IKA, Königswinter, Germany). Lymphocytes were separated using a 40% Percoll solution and gradient centrifugation. The supernatant and lipid layer were discarded, and the cells were washed twice with DPBS. Red blood cells were lysed using BD Pharm Lyse™ Lysing Buffer (Becton Dickinson and Company, Franklin Lakes, NJ, USA), leaving nonparenchymal cells. The spleen was removed and ground into a cell suspension, which was filtered into a single-cell suspension using a 70-μm cell sieve (Becton Dickinson). Red blood cells were lysed, and the remaining cells were washed twice with 1× DPBS to obtain splenic lymphocytes.

### Isolation of Natural Killer Cells From Lymphocytes

The concentration of hepatic non-parenchymal cells (NPC) was adjusted to 1×10^7^ cells. An NK Cell Isolation Kit (Miltenyi Biotec, Auburn, CA, USA) was used to isolate NK cells. The cell suspension was centrifuged, and the supernatant was thoroughly removed. The cell pellet was resuspended in 40 µl of the buffer, 10 µl of NK Cell Biotin-Antibody Cocktail per 10⁷ total cells was added, and incubated for 5 min in the refrigerator. After washing, 80 µl of buffer and 20 µl of Anti-Biotin MicroBeads per 10⁷ cells were added, and cells were incubated. After washing, the cell suspension was applied onto the column. The flow-through containing unlabeled cells was collected, representing the enriched NK cells.

### Flow Cytometry

Liver non-parenchymal cells and splenic lymphocytes were adjusted to 1 × 10^6^/ml using fluorescence-activated cell sorting (FACS) buffer (2% bovine serum albumin and 2 mM ethylenediaminetetraacetic acid in DPBS). The following antibodies were used in our experiments: phycoerythrin (PE)-cy7-conjugated anti-mouse NK1 homeobox 1 (NK1.1; clone: PK136); peridinin chlorophyll protein complex (PerCP)-cy5.5-conjugated anti-mouse CD11b (integrin subunit alpha M; M1/70); fluorescein isothiocyanate (FITC)-conjugated anti-mouse CD11b (integrin subunit alpha M; M1/70); FITC-conjugated anti-mouse Ly49 (killer cell lectin-like receptor subfamily A, member 1; 5E6); Brilliant Violet 421 (Bv421)-conjugated anti-mouse NKp46 (natural killer cell P46-related protein; 29A1.4); PE-conjugated anti-mouse NKG2D (CX5); allophycocyanin (APC)-conjugated anti-mouse NKG2A (natural killer group protein 2A); PE-conjugated anti-mouse Immunoglobulin G (IgG1; R3-34); and APC-conjugated anti-mouse IgG2a (R35-95). All of these antibodies were purchased from BD Biosciences (San Jose, CA, USA). NK cells were defined as CD11b^+^NK1.1^+^. Ly49, NKp46, NKG2A, and NKG2D are receptors of NK cells. Cells were stained with different combinations of antibodies for 30 min at room temperature in the dark, followed by one wash with FACS buffer. All experiments were performed using a BD FACS Verse flow cytometer (BD Biosciences). Data were analyzed using FlowJo 10 software (TreeStar Inc., Ashland, OR, USA).

### Cytokine Detection Using a Cytometric Bead Array

Cytokines in the sera of uninfected mice and mice infected with *S. japonicum* at 2 to 10 weeks post-infection were detected by using a cytometric bead array (CBA). The contents of one albumin standard (BSA) ampule were diluted into eight standards and one blank. Each standard and the samples were mixed with a suitable concentration of microspheres and incubated at room temperature for 1 h. PE-conjugated anti-mouse cytokine-detection antibodies were added to the microspheres and incubated at room temperature for 1 h in the dark. Samples were washed twice and suspended in wash buffer for measurement using the Accuri C6 system (BD Biosciences).

### Quantitative Real-Time Reverse Transcription-PCR

Total RNA from NK cells was extracted using an miRNeasy Mini Kit (Qiagen, Hilden, Germany) from uninfected mice and mice at 2 to 10 weeks post-infection. Complementary DNA (cDNA) was synthesized using 500 ng of total RNA with a Prime Script RT Master Mix (Takara, Shiga). Quantitative real-time reverse transcription**-**PCR (qRT-PCR) was used to detect the expression of genes, including those encoding IFN-γ, granzyme b, and perforin using Fast SYBR Green master Mix (Bio-Rad, Hercules, CA, USA). Total RNA from mice livers were extracted, and cDNA was synthesized. The expression of genes, including those encoding collagen I, collagen I, and α-SMA were also detected by qPCR with a CFX96 real-time system (Bio-Rad, Hercules, CA, USA). Data were analyzed as relative messenger RNA (mRNA) expression and were normalized to the mRNA level of glyceraldehyde-3-phosphate dehydrogenase (*Gapdh*).

### Histopathological Evaluation of Liver Fibrosis

The right lobes of the livers from uninfected mice and from mice at 2 to 10 weeks post-infection was fixed in paraformaldehyde. Liver tissues were dehydrated, embedded in paraffin, and then cut into 4-μm slices for hematoxylin and eosin (HE) and Masson staining. Slices stained with HE was examined under a light microscope to observe the formation and changes in egg granulomas. Slices with Masson staining were examined to observe the changes in collagen deposition, which exhibits a blue-stained area.

### Statistical Analyses

All data were analyzed using SPSS20.0 software (IBM Corp., Armonk, NY, USA). Differences between groups were assessed using nonparametric one-way analysis of variance. Values in the text are presented as the means ± standard deviation (SD). *P* < 0.05 indicated a significant difference.

## Results

### The Profiles of Natural Killer Cells in the Liver and Spleen of *Schistosoma japonicum*-Infected Mice

**Figure 1:**
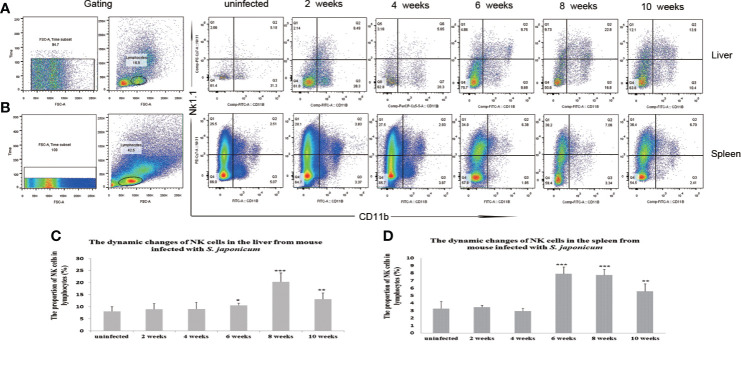
Dynamic changes in the proportions of natural killer (NK) cells in the liver and spleen after *Schistosoma japonicum* infection. The proportions of NK cells increased from 6 to 10 weeks post-infection in the liver and spleen of *S. japonicum*-infected mice. **(A)** Scatter diagrams of the proportions of NK cells in lymphocytes from the liver. **(B)** Scatter diagrams of the proportions of NK cells in lymphocytes from the spleen. **(C)** The dynamic changes of the proportions of NK cells in the liver. **(D)** The dynamic changes of the proportions of NK cells in the spleen. *p < 0.05, **p < 0.01, ***p < 0.001. Results were repeated for three times, and the number of mice was not less than four per time point in each time.

The proportion of NK cells in the liver and spleen increased post-infection. The proportions of NK cells in lymphocytes from the liver were 10.56 ± 0.87, 20.37 ± 3.7, and 13.18 ± 2.56% from 6 to 10 weeks post-infection (sampled every 2 weeks), which were significantly higher than that in the uninfected group at 8.11 + 1.89% (P< 0.05). The proportions of NK cells in lymphocytes in the spleen were 7.91 ± 0.92, 7.56 ± 0.74, and 5.58 ± 0.97% from 6 weeks to 10 weeks post-infection, which were significantly higher than that in the uninfected group at 3.28 ± 0.95% (*P* < 0.05) (**Figure 1** and **Supplementary Figures S1**–**S3**).

### Changes of Inhibitory Receptors and Activated Receptors on Natural Killer Cells From Liver and Spleen

**Figure 2:**
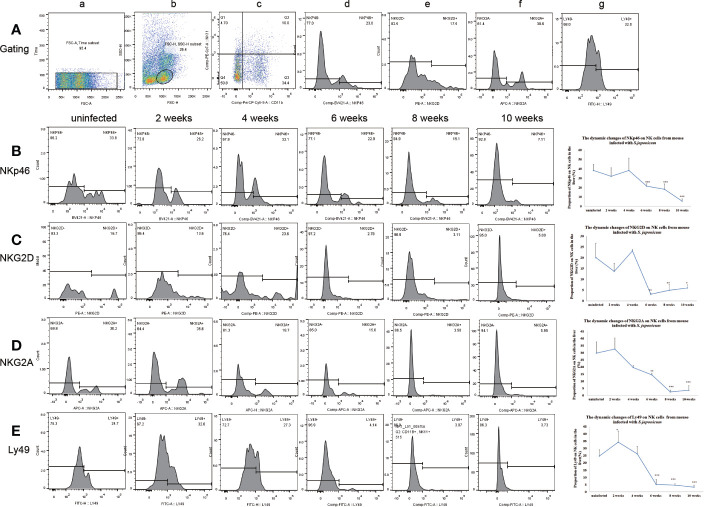
Expression of receptors on natural killer (NK) cells in the livers from mice infected with *Schistosoma japonicum*. **(A)** Gating strategy of hepatic NK cells; a. time gate, b. lymphocytes, c. CD11b^+^NK1.1^+^NK cells, d. gating of NKp46 on the surface of NK cells, e. gating of NKG2D on the surface of NK cells, f. gating of NKG2A on the surface of NK cells, g. gating of Ly49 on the surface of NK cells. **(B)** Changes in NKp46 expression on NK cells among the lymphocytes. a. from an uninfected mouse, b. from a mouse at 2 weeks post-infection, c. from a mouse at 4 weeks post-infection, d. from a mouse at 6 weeks post-infection, e. from a mouse at 8 weeks post-infection f. from a mouse at 10 weeks post-infection. **(C)** Changes in NKG2D expression on NK cells among the lymphocytes. a. from an uninfected mouse, b. from a mouse at 2 weeks post-infection, c. from a mouse at 4 weeks post-infection, d. from a mouse at 6 weeks post-infection, e. from a mouse at 8 weeks post-infection f. from a mouse at 10 weeks post-infection. **(D)** Changes in NKG2A expression on NK cells among the lymphocytes. a. from an uninfected mouse, b. from a mouse at 2 weeks post-infection, c. from a mouse at 4 weeks post-infection, d. from a mouse at 6 weeks post-infection, e. from a mouse at 8 weeks post-infection f. from a mouse at 10 weeks post-infection. **(E)** Changes in Ly49 expression on NK cells among the lymphocytes. a. from an uninfected mouse, b. from a mouse at 2 weeks post-infection, c. from a mouse at 4 weeks post-infection, d. from a mouse at 6 weeks post-infection, e. from a mouse at 8 weeks post-infection f. from a mouse at 10 weeks post-infection. NK, natural killer cell; Ly49, killer cell lectin-like receptor subfamily A, member 1; NKG2A, natural killer group protein 2A; NKp46, natural killer cell P46-related protein; NKG2D, natural killer group protein 2D; ^*^p<0.05, ^**^p<0.01, ^***^p<0.001. Results were repeated for three times, and the number of mice was not less than four per time point in each time.

**Figure 3:**
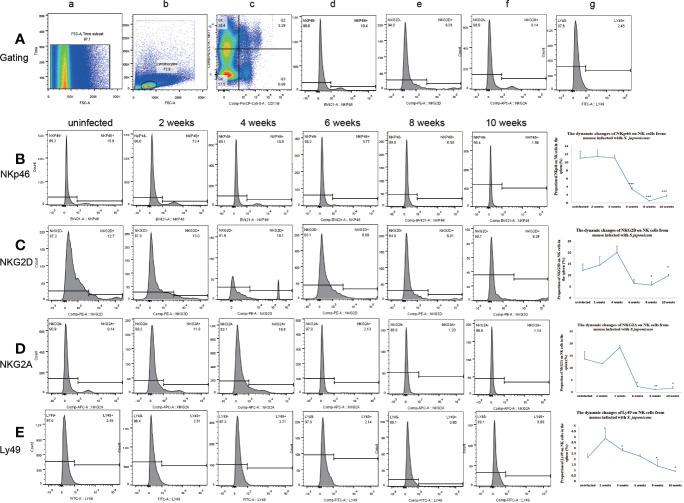
Expression of receptors on natural killer (NK) cells in the spleen from mice infected with *Schistosoma japonicum*. **(A)** Gating strategy of splenic NK cells; a. time gate, b. lymphocytes, c. CD11b^+^NK1.1^+^NK cells, d. gating of NKp46, e. gating of NKG2D, f. gating of NKG2A, g. gating of Ly49. **(B)** Changes in NKp46 expression on NK cells among the lymphocytes. a. from an uninfected mouse, b. from a mouse at 2 weeks post-infection, c. from a mouse at 4 weeks post-infection, d. from a mouse at 6 weeks post-infection, e. from a mouse at 8 weeks post-infection f. from a mouse at 10 weeks post-infection. **(C)** Changes in NKG2D expression on NK cells among the lymphocytes. a. from an uninfected mouse, b. from a mouse at 2 weeks post-infection, c. from a mouse at 4 weeks post-infection, d. from a mouse at 6 weeks post-infection, e. from a mouse at 8 weeks post-infection f. from a mouse at 10 weeks post-infection. **(D)** Changes in NKG2A expression on NK cells among the lymphocytes. a. from an uninfected mouse, b. from a mouse at 2 weeks post-infection, c. from a mouse at 4 weeks post-infection, d. from a mouse at 6 weeks post-infection, e. from a mouse at 8 weeks post-infection f. from a mouse at 10 weeks post-infection. **(E)** Changes in Ly49 expression on NK cells among the lymphocytes. a. from an uninfected mouse, b. from a mouse at 2 weeks post-infection, c. from a mouse at 4 weeks post-infection, d. from a mouse at 6 weeks post-infection, e. from a mouse at 8 weeks post-infection f. from a mouse at 10 weeks post-infection. ^*^p < 0.05, ^**^p < 0.01, ^***^p < 0.001. Results were repeated for three times, and the number of mice was not less than four per time point in each time.

The expression of Ly49 on NK cells increased significantly at 2 weeks post-infection in the liver and spleen. Ly49 expression decreased from 6 to 10 weeks post-infection in the liver and from 8 to 10 weeks post-infection in the spleen. The expression of NKG2A and NKp46 on NK cells decreased from 6 to 10 weeks post-infection in the liver and spleen. The expression of NKG2D on NK cells decreased significantly from 6 to 10 weeks post-infection in the liver and from 8 to 10 weeks in the spleen. (**Figures 2** and **3** and **Supplementary Figures S4**–**S7**). In conclusion, the expression of both inhibitory and activated receptors on NK cells in the liver and spleen decreased significantly from 6 or 8 weeks to 10 weeks post-infection. Only Ly49 expression increased at 2 weeks post-infection in the liver and spleen.

### Changes in Effector Molecules of Natural Killer Cells From Infected Mice

**Figure 4:**
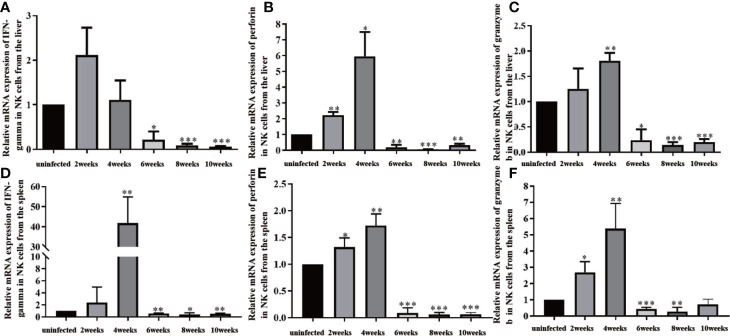
Changes in effector molecules of natural killer (NK) cells in the liver and spleen of infected mice. **(A)** Changes in IFN-gamma expression in NK cells isolated from the liver. **(B)** Changes in perforin expression in NK cells isolated from the liver. **(C)** Changes in granzyme b expression in NK cells isolated from the liver. **(D)** Changes in IFN-gamma expression in NK cells isolated from the spleen. **(E)** Changes in perforin expression in NK cells isolated from the spleen. **(F)** Changes in granzyme b expression in NK cells isolated from the spleen. ^*^p < 0.05, ^**^p < 0.01, ^***^p < 0.001. Results were repeated for three times, and the number of mice was not less than four per time point in each time.

NK cells were isolated from liver and spleen lymphocytes using magnetic beads. From 2 to 4 weeks post-infection, levels of IFN-γ, granzyme b, and perforin in NK cells from the liver and spleen increased significantly. These effector molecules peaked at 4 weeks post-infection. From 4 to 10 weeks post-infection, the levels of IFN-γ, granzyme b, and perforin in NK cells from the liver decreased, and the level of IFN-γ in NK cells from the spleen decreased significantly (**Figure 4**). The above results indicated that NK cells in the liver were activated from 2 to 4 weeks, and inhibited from 6 to 10 weeks post-infection.

### Changes in Cytokines in the Sera of Infected Mice

**Figure 5:**
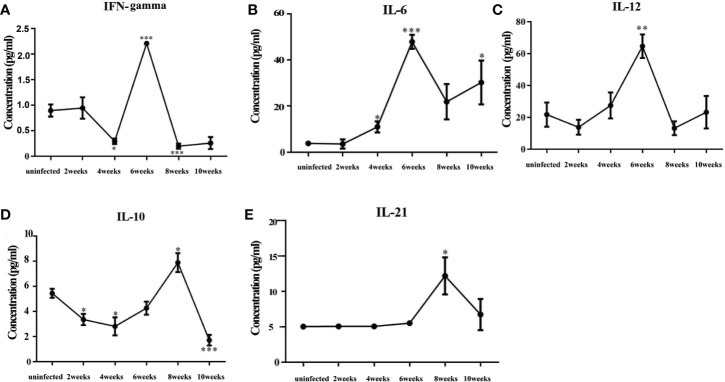
Changes in cytokines in the sera of mice infected with *Schistosoma japonicum*. **(A)** The levels of IFN-γ in mouse sera. **(B)** The levels of IL-6 in mouse sera. **(C)** The levels of IL-12 in mouse sera. **(D)** The levels of IL-10 in mouse sera. **(E)** The levels of IL-21 in mouse sera. The levels of inflammatory factors, such as IFN-γ, IL-6, and IL-12, peaked at 6 weeks after infection. The levels of IL-10 and IL-21 peaked at 8 weeks after infection. IFN-γ, interferon-gamma; IL-6, interleukin 6; IL-12, interleukin 12; IL-10, interleukin 10; IL-21 interleukin 21. ^*^p < 0.05, ^**^p < 0.01, ^***^p < 0.001. Results were repeated for three times, and the number of mice was not less than four per time point in each time.

The levels of inflammatory cytokines in mouse sera, such as IFN-γ, IL-6, and IL-12, peaked at about 6 weeks post-infection, which indicated that the mice had mounted an acute inflammatory response (**Figure 5**). Cytokines decreased sharply at 8 weeks post-infection, while the levels of IL-10 and IL-21 increased significantly. These results showed that the mice were undergoing inflammatory repair at about 8 weeks post-infection.

### Hepatic Fibrosis Evaluation

**Figure 6:**
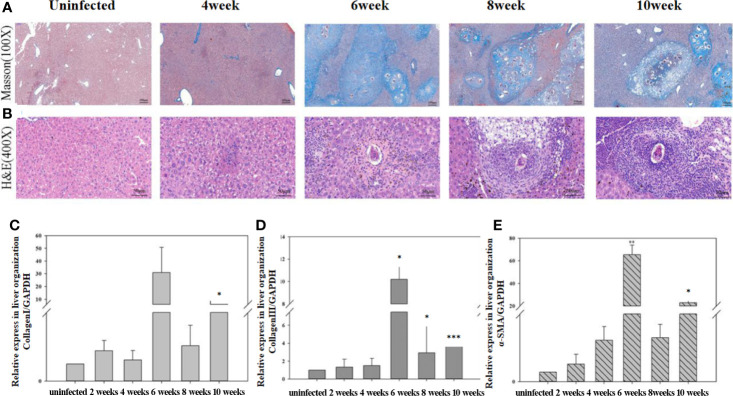
The progression of hepatic fibrosis in mice after infected with *Schistosoma japonicum*. **(A)** Liver tissue stained using Masson staining at different time points after infection. **(B)** Liver tissue stained using hematoxylin-eosin (HE) at different time points after infection. **(C)** The expression of collagen I in liver detected using quantitative real-time reverse transcription PCR (qRT-PCR). **(D)** The expression of collagen III in liver detected using qRT-PCR. **(E)** The expression of α-SMA in the liver detected using qRT-PCR. The levels of collagen I, collagen III, and α-SMA in the liver increased significantly from 6 to 10 weeks post-infection compared with that before infection. The highest expression level occurred at 6 weeks post-infection. The results of HE and Masson staining revealed a significant increase in collagen deposition from 6 weeks post-infection. ^*^p < 0.05, ^**^p < 0.01, ^***^p < 0.001. Results were repeated for three times, and the number of mice was not less than four per time point in each time.

The expression levels of the mRNAs encoding α-SMA, collagen I, and collagen III in the liver were detected using qRT-PCR (**Figure 6**). The staining results showed that the liver structure was basically normal at 4 weeks post-infection. At 6 weeks post-infection, many acute egg granulomas had formed, and a large amount of lymphocyte infiltration was observed. Egg granulomas fused, collagen deposition increased significantly, and liver fibrosis was observed at 8 to 10 weeks post-infection. The results of qRT-PCR showed that the expression of α-SMA, collagen I, and collagen III mRNAs increased significantly from 6 to 10 weeks post-infection compared with the mice in the uninfected control group. The highest expression levels of α-SMA, collagen I, and collagen III mRNAs were observed at 6 weeks post-infection.

## Discussion

After *S. japonicum* maturated and laid eggs, the schistosome eggs were deposited in the host liver and released SEA. The inflammatory response induced by SEA prompted various immune cells to secrete a large number of cytokines and activated molecules. These chemical signals worked together to activate the resting HSC to differentiate into myofibroblasts, which then secreted a large number of collagen fibers (Carson et al., 2018). When the rate of collagen synthesis exceeded that of degradation, liver fibrosis begins to occur (Zhang et al., 2016). Many immune cells, such as NK cells (Hou et al., 2012; Fasbender et al., 2016), natural killer T cells (NKT) (Mitra et al., 2014), and macrophages (Ma et al., 2017; Tsuchida and Friedman, 2017), could inhibit HSC cell activation and play an anti-fibrotic role, among which the anti-fibrotic effect of NK cells was the clearest. In the *S. japonicum* infection model, NK could negatively regulate egg-induced liver fibrosis by producing IFN-γ (Hou et al., 2012). A similar phenomenon was found in an *S. mansoni* infection model (Czaja et al., 1989).

NK cells are critical immune cells in innate immunity, which exert an anti-liver fibrosis effect. NK cells in mice express many essential markers, such as CD11b (MAC-1) and NK1.1, during the development of NK cells. CD11b is an essential marker of NK cell maturation (Hayakawa et al., 2010). In Hayakawa’s research, mouse NK1.1^+^CD3^−^NK cells could be divided into CD11b^lo^ and CD11b^hi^ populations in the spleen, liver, lung, bone marrow, and lymph node. CD11b^hi^ NK cells were significantly more mature than CD11b^lo^ NK cells (Hayakawa et al., 2010). In our research, NK1.1^+^CD11b^+^ was used to define mature NK cells. Due to the experimental operation, we were not sure that the number of liver parenchymal cells would be the same each time. However, we still observed that the proportions of NK1.1^+^CD11b^+^ NK cells in the liver and spleen were significantly increased at 6 to 10 weeks post-infection.

Many activated receptors, such as NKp46, NKG2D, and 2B4; and inhibitory receptors, such as NKG2A, Ly49, and Tim3, are expressed on NK cells (Grudzien and Rapak, 2018). The integration of all the signals through the receptors strictly regulates NK cell behavior and ultimately determines the degree of cytotoxicity and cytokine production mediated by NK cells (Orr and Lanier, 2010; Long et al., 2013; Narni-Mancinelli et al., 2013; Watzl, 2014). NK cell surface activated receptor NKG2D could bind to the activated HSC cell surface retinoic acid early transcript 1 (rae-1) to trigger the killing of HSC cells in coordination with soluble MHC I-like molecular related protein A (Krizhanovsky et al., 2008; Fasbender et al., 2016). NK cells could also express TNF-related apoptosis-inducing ligands (TRAILs) and other death signals (Li et al., 2019) or release IFN-γ and IL-22 (Kong et al., 2012), activate the STAT1 signaling pathway, and induce HSC apoptosis or cell cycle arrest (Tosello-Trampont et al., 2017). Activated HSCs could also downregulate the expression of MHC-I, and induce NK cell killing of HSCs (Luo et al., 2014; Mitra et al., 2014). The population of NK cells (NK1.1^+^CD11b^+^) in the liver increased significantly from 6 to 10 weeks after infection in the liver and spleen. However, the NK cells did not inhibit the formation of hepatic fibrosis, which might be related to the functional state of the NK cells. At 2 weeks after infection, only Ly49 expression increased significantly. The Ly49 receptor family in mice includes Ly49A, Ly49C, Ly49G2, and Ly49F (Rahim and Makrigiannis, 2015). It was not easy to judge whether the receptor was acting as an IR or an AR. The expression levels of NKG2D, NKp46, NKG2A, and Ly49 on NK cells decreased at 6 to 10 weeks post-infection. There are many activating and inhibitory receptors on the surface of NK cells. In our study, we selected four more important receptors, but they showed a trend of decreasing expression at 4 weeks after infection. In the BALB/c mouse model of liver fibrosis induced by *S. japonicum* infection, it is very important to further screen highly expressed inhibitory receptors of NK cells to assess the inhibitory state of the NK cells.

Levels of IFN-γ, granzyme b, and perforin in NK cells in the liver increased from 2 to 4 weeks post-infection, and decreased significantly from 6 to 10 weeks post-infection. These results suggested NK cells became activated at some point before 4 weeks post-infection and were inhibited after 6 weeks post-infection. Thus, NK cell function changed from 4 to 6 weeks post-infection, when *S. japonicum* proliferated, matured, and began to lay eggs. The inhibition of NK cells might be related to SEA. Some reports showed that in tumors and chronic infection, NK cells were inhibited, which was characterized by decreased secretion of effector factors (such as IFN-γ) and decreased expression of ARs (Bi and Tian, 2017; Liu et al., 2018). Our research showed that NK cells were also inhibited after massive deposition of eggs in the liver.

Cytokines in serum reflect the immune status of the body as a whole. Our results showed that the levels of inflammatory cytokines, such as IFN-γ, IL-6, and IL-12, in the sera of infected mice peaked at about 6 weeks post-infection, which showed that the mice were undergoing an inflammatory response (Hamidzadeh et al., 2017). The levels of inflammatory cytokines decreased sharply at about 8 weeks post-infection, at which time the levels of IL-10 and IL-21 peaked. IL-21 promotes the proliferation and differentiation of B cells and the class transformation of antibodies (Davis et al., 2015). Previous research showed that IL-10 reduced inflammation and promoted the repair process (Bodaan et al., 2016). Some reports showed that after being infected with *S. mansoni*, high levels of IL-10 could regulate the frequency and intensity of allergic reactivity (Resende et al., 2019). We concluded that at about 8 weeks post-infection, the mice were engaged in repairing the damage caused by inflammation. The expression of α-SMA, collagen I, and collagen III increased significantly from 6 to 10 post-infection, which also suggested active repair of inflammation-related damage. Some reports showed that after egg deposition in the liver, pro-fibrotic factors, such as VEGF, IL-13, TGF-β, or IL-33, also increased and promoted the formation of liver fibrosis (Costain et al., 2018). In our experiment, cytokine values, especially IFN-γ, IL-10, and IL-21, were not particularly high, which may be related to the low number of cercariae infection.

After *S. japonicum* began to lay their eggs, the immune response might be reversed significantly in the mice. Before *Schistosoma* laid their eggs, the host was in an inflammatory phase. The levels of inflammatory cytokines (IFN-γ, IL-6, and IL-12) in sera increased, in which activated NK cells contributed partly to this process. After *Schistosoma* was laid their eggs, the inflammatory response in the host was gradually suppressed and the damage caused by inflammation was gradually repaired. The proportion of NK cells increased; however, the function of NK cells gradually became inhibited. Negative regulator IL-10 and the B cell proliferation-promoting factor IL-21 peaked at this time. In addition, liver fibrosis gradually formed.

A previous study showed that NK cells have an anti-fibrotic effect in schistosomiasis models by killing HSC-derived myofibroblasts (Hou et al., 2012). In our research, we found that from 6 to 10 weeks post-infection, the functions of NK cells were inhibited, with significantly decreased levels of activated receptors, inhibitory receptors, and effector molecules. We concluded that NK cells became disabled during this time. Reversing the inhibition of liver NK cells might represent a strategy to inhibit the progress of liver fibrosis.

## Data Availability Statement

The original contributions presented in the study are included in the article/supplementary materials, further inquiries can be directed to the corresponding authors.

## Ethics Statement

The animal study was reviewed and approved by the LAWEC Committee of the National Institute of Parasitic Diseases, Chinese Centre for Disease Control and Prevention.

## Author Contributions

YH and JC conceived and designed the experiments. YH, XW, YW, and JZ performed the experiments. YH, YS, HL, and JC analyzed the data. YH and JC contributed reagents and materials. YH and JC wrote and revised the manuscript. All authors contributed to the article and approved the submitted version.

## Funding

This work was supported by the Major National Science and Technology Projects (No. 2018ZX10102001-002-004 to YH), the Shanghai Natural Science Foundation (No. 19ZR1462600 to YH), and the National Nature Science Foundation of China (Nos. 8177225 and 81971969 to JC). The funders had no role in the study design, data collection, and analysis, decision to publish, or preparation of the manuscript.

## Conflict of Interest

The authors declare that the research was conducted in the absence of any commercial or financial relationships that could be construed as a potential conflict of interest

## Supplementary Material

Supplementary Figure 1 Time gate strategy of all samples. The time gate was checked before analyzing the sample to ensure the stability of the data.

Supplementary Figure 2 Anti-mouse CD11b staining controls.

Supplementary Figure 3 Anti-mouse NK1.1 staining controls.

Supplementary Figure 4 Anti-mouse NKG2D staining controls.

Supplementary Figure 5 Anti-mouse NKG2A staining controls.

Supplementary Figure 6 Anti-mouse Ly49 staining controls.

Supplementary Figure 7 Anti-mouse NKp46 staining controls.
